# Commentary: A Machine-Generated View of the Role of Blood Glucose Levels in the Severity of COVID-19. A Metabolic Endocrinology Perspective

**DOI:** 10.3389/fendo.2022.877973

**Published:** 2022-04-28

**Authors:** Jeff M. P. Holly

**Affiliations:** Faculty of Medicine, School of Translational Health Science, Bristol Medical School, University of Bristol, Southmead Hospital, Bristol, United Kingdom

**Keywords:** COVID-19, hyperglycemia, obesity, diabetes, metabolism, public health, mucormycosis

## Introduction

Endocrinologist are well aware of the many adverse health issues consequent to the global pandemic of metabolic disturbances, such as obesity and diabetes, in which hyperglycemia plays a central role. In the years immediately prior to the COVID-19 pandemic a number of health experts warned that the global diabetes pandemic would have serious implications for the burden of infectious diseases in low- and middle-income countries (1). The implications of diabetes were soon apparent when, in 2019, COVID-19 became the first global infectious disease pandemic in a century. However, it also became evident that many of the low- and middle-income countries generally did much better than some of the richest nations and that global perspectives of public health had been turned upside-down (2). The diabetes and obesity pandemics have had a major impact on the global burden inflicted by COVID-19; with many reporting that these metabolic disorders increased the severity, morbidity and mortality due to COVID-19 (3, 4). In contrast to the prior coronavirus epidemics of SARS and MERS, the severity of disease became a critical issue for COVID-19 and presented a huge challenge to public health as many infections were asymptomatic, or only led to minor symptoms: this resulted in much greater transmission and the rapid global spread of COVID-19. As a consequence of this, even though only a small proportion of cases progress to severe disease, the huge numbers of cases has resulted in millions of deaths globally. In order to address the factors critical to the progression from mild to severe life-threatening disease, scientists working on the Blue Brain Project in Lausanne, Switzerland applied their powerful computers and expertise in Artificial Intelligence (AI) to interrogate the vast literature published on COVID-19 to detect common linked threads (5). The resultant manuscript described many new insights into the effects of hyperglycemia in the body; described within an encyclopedic review with 422 citations, 26 figures, 2 tables and multiple supplements. This perspective summarises the implications of their conclusions for endocrinologists and also highlights how this may inform some of the other concerns for metabolic endocrinologists that have emerged from the COVID-19 pandemic.

## AI Synthesis of the Literature on the Determinants of COVID-19 Severity

Using machine reading and knowledge engineering tools the Swiss group mined the information from 240,000 published articles that were openly accessible in the CORD-19 database and condensed the common information into a unified hypothesis for why COVID-19 took on a more severe clinical course in certain individuals (5). The authors developed entity extraction and linking applications to mine and extract structured information from this vast number of scientific publications and create a knowledge graph to synthesize and navigate the knowledge into an integrated pathway. They also developed knowledge engineering tools that then generated a unifying hypothesis. This proposed that hyperglycemia was the single most critical factor that led to more severe disease in specific cases. This was complimented with a further computer tool that was developed, termed a BioExplorer, that reconstructed at an atomistic level the virus and its immediate environment at the surface of the pulmonary airways to model the effects of hyperglycemia on the initial steps of infection (5).

The machine learning knowledge synthesis identified that hyperglycemia affects a panoply of mechanisms that determine how the body responds to a coronavirus infection. Such as a weakened immune system, a chronic proinflammatory and atherothrombotic state and the accumulation of advanced glycation end products, as familiar to most endocrinologists (3, 4). Furthermore, the ‘machines’ revealed hyperglycemia has remarkably extensive additional effects reducing the bodys’ defences at every level and facilitating every step of the life cycle of the SARS-CoV-2 virus thus aggravating the severity of the infection. These effects started with the initial innate defences in the airways with high glucose levels in the airway surface liquid (ASL), consequent from systemic hyperglycemia, resulting in acidification of the ASL, reducing the mucociliary clearance capacity, reducing the lectin-mediated recognition of the virus, reducing the general activity of antimicrobial agents in the airways and the number and activity of resident macrophages and neutrophils (5). Furthermore, the BioExplorer modelling generated further intriguing possibilities of how a high glucose environment could promote viral entry and replication in host cells in addition to affecting the host immune response: culminating in a cytokine storm leading to acute respiratory distress syndrome and ultimately the multiorgan failure (5). These effects would be primed by pre-existing diabetes. In addition, the inflammation and critical illness associated with COVID-19, and frequently treated with high-dose glucocorticoids, could all cause hyperglycemia in those without prior diabetes which could also promote disease progression *via* the mechanisms revealed by AI.

In addition to the remarkable feat of assimilating a more complete picture of how hyperglycemia can promote the severity of COVID-19, the review raised numerous questions for future research to address. One of the most immediate questions being whether existing therapeutics for diabetes could be beneficial for treating, or even preventing, severe COVID-19. The evidence from observational studies are heavily confounded by the different medications being introduced at different stages throughout the pathway for the clinical management of diabetes. First-line drugs, such as metformin, are generally introduced to manage early diabetes and subsequently more aggressive and more combinations of drugs are then added as the diabetes progresses and eventually insulin is used when all pancreatic reserve is lost. Retrospective comparisons of clinical outcomes of patients who were infected with COVID-19 generally revealed that patients on first-line drugs, such as metformin, did better than those on medications used for more advanced diabetes and this was extracted by the AI. However, this probably reveals little more than indicate that patients with mild diabetes do better with infections than those with more severe diabetes (6). Whether there are any benefits of diabetes drugs for preventing or managing COVID-19 will depend on prospective intervention trials and there are already many such clinical trials now examining this question. The answer to the immediate question of how to manage metabolic control in patients with severe COVID-19 is clearer: as with any critically ill patients the consensus is to use intravenous insulin with continuous glucose monitoring, preferably using a closed-loop device (7, 8). There have been some concerns raised that insulin therapy may increase mortality due to COVID-19 (9, 10). This may partly be due to those with existing diabetes who were already treated with insulin were those with the most severe diabetes. However, glucose management with insulin in critically ill patients, who have inflammation, insulin-resistance and are frequently treated with high-dose glucocorticoids, is notoriously difficult (11), particularly at the height of a pandemic when staff with intensive care experience may be stretched.

## Further Collateral Challenges for Endocrinologists

There are many further challenges that endocrinologists face in managing metabolic disease as a consequence of COVID-19 and indeed the pandemic has had many adverse metabolic effects that are independent of those directly related to COVID-19: so-called collateral damage. With health services overrun by COVID-19 there was reduced access to both primary and secondary care facilities. A large study of primary care records in the UK, comparing the initial pandemic period with trends from the previous 10 years, found a 70% reduction in new diagnoses of type 2 diabetes, a 77% reduction in HbA1c testing and a 53% reduction in new prescriptions for metformin and it was estimated that there were 60,000 missed or delayed diagnoses of type 2 diabetes across the UK (12). Such delay in diagnoses or escalation of clinical care could increase the risk of future avoidable diabetes complications. Further to the reduced access to clinical care, stay-at-home and ‘lock-down’ restrictions resulted in many having reduced physical activity, reduced quality of nutrition, reduced quality of sleep, increased alcohol consumption, increased stress and reduced exposure to sunlight with potential reduction of Vitamin D levels: all of which would have led to weight gain and aggravated metabolic control. In another population-based study in the UK, looking at excess deaths in patients with diabetes, comparing the period across the first wave of COVID-19 in the UK with the corresponding period from the previous 3 years, it was found that there were not only considerable additional COVID-19-related deaths but also a 15.8-22.2% increase in deaths that were not directly related to COVID-19 (13). COVID-19 has accelerated the global pandemic of hyperglycemia. A panel of international experts has recently been established to advise on the additional challenges for managing metabolic diseases during the pandemic (14).

Within the global COVID-19 pandemic there have also emerged two further associated pandemics. Post-acute sequelae of severe acute respiratory syndrome coronavirus 2 infection (PASC), or long-COVID, is a global problem that will present considerable additional long-term burdens on health systems. Murcomycosis is a more limited and more regional issue but which incurs very serious additional morbidity. Evidence is accumulating indicating that hyperglycemia may be a factor in both of these pandemics within the main COVID-19 pandemic and the further insights into the panoply of effects of hyperglycemia that were revealed by AI (5) may broaden our understanding of both.

## Should New-Onset Diabetes Be Considered Among the Many Symptoms of Long-COVID?

The definition of long-COVID is still being agreed with the National Institute for Health and Care Excellence in the UK proposing symptoms continuing for over 12 weeks (15) and the World Health Organization (WHO) proposing the definition as a range of symptoms occurring 3 or more months after confirmed SARS-CoV-2 infection that last for at least 2 months and that generally have an impact on daily functioning (16). Although estimates of the prevalence of long-COVID vary, at least partly due to differences in populations and definitions, the emerging picture indicates that the chronic sequalae will incur considerable burden on health care systems and significant costs to society (17). A multicenter study of patients admitted to hospital for COVID-19 in the UK found that 20% had acquired a new disability at a median of 5 months following discharge from hospital and 19% had experienced a health-related change in occupation (18). Similarly, a much larger healthcare database study in the USA, found that overall 7% experienced symptoms 6 months after the acute disease, but this rose to 21.7% in those that had been initially hospitalized and to 36% in those that were admitted to Intensive Care Units (ICU) (19). With over 271 million COVID-19 cases in the world as of 16^th^ December 2021 (https://covid19.who.int/), which represents 3.5% of the global population, this indicates that long-COVID will be a very large long-term burden on society. The etiology of long-COVID symptoms is still not clear but it has been suggested to be a form of post-sepsis syndrome (20, 21) and autoimmunity has been proposed to play a role (22).

There has been concern that one of the sequalae of long-COVID will be an increase in metabolic disorders, including new cases of diabetes. The adverse effects of infection and critical illness on metabolic control are well established (23). There have been many retrospective follow-up cohort studies of COVID-19 cases that have generally reported an increase in the severity of existing diabetes and in the incidence of new-onset diabetes in the post-acute phase (24–30). There has also been a bidirectional Mendelian randomization study that established a potential causal effect of COVID-19 on the subsequent risk of new-onset type 2 diabetes (31). These reports of new-onset diabetes in the post-acute phase would fall within the WHO definition of long-COVID if the diabetes were a direct consequence of COVID-19. In addition, there have been a number of suggestions that COVID-19 may be a trigger for the onset of type 1 diabetes in children. A multicentre study from London reported at 80% increase in type 1 diabetes cases during the pandemic compared to the previous 5 years and suggested that COVID-19 may precipitate or accelerate type 1 diabetes onset in children (32). Consistent with a potential direct effect of COVID-19 causing pancreatic damage several studies have reported the presence of SARS-CoV-2 and its cell receptor, angiotensin-converting enzyme 2 (ACE2), in pancreatic beta cells, along with evidence of fibrosis associated with multiple vascular thrombi in autopsy tissue obtained from patients who had died from COVID-19 (33–35). In addition, there have been several laboratory studies demonstrating that SARS-CoV-2 can infect and damage pancreatic beta-cells in culture (33, 35, 36). These potential effects on the pancreas were highlighted by the AI (5). However, the role that virus-induced pancreatic beta-cell damage plays in causing new diabetes is still debated (37, 38).

It is clear that the relationship between hyperglycemia and COVID-19 is a bidirectional link; not only does hyperglycemia promote a more severe acute COVID-19, but COVID-19 can aggravate metabolic control. As with the acute infection, there may be a bidirectional relationship between long-COVID and metabolic disturbances with evidence that diabetes and obesity can increase the risk of developing long-COVID. Although long-COVID was originally thought to be only weakly related to the severity of the initial COVID-19 disease (39); the emerging evidence indicates that the prevalence of long-COVID increases considerably in those that initially needed hospitalisation and increase further in those admitted to ICU (19). As metabolic disturbance appears to be the critical factor in facilitating more severe COVID-19 (5) and long-COVID is more prevalent in those with severe disease, this implies that metabolic dysregulation would increase the risk of long-COVID. Obesity has also been reported to be associated with a greater number of long-term post-COVID symptoms (40).

Despite all of these reports there remain many questions regarding whether COVID-19 can actually cause diabetes as there a number of important potential confounding issues. The many side-effects of lock-downs, as described above, may have promoted or precipitated the progression to diabetes in subjects with pre-diabetes and there may also be catch-up of the many missed diagnoses at the height of the pandemic. An even greater confounder relates to the evidence that throughout the world more than 50% of people with diabetes remain undiagnosed (41) and the increased testing may have led to many undiagnosed diabetes cases being recognized, inflating the apparent incidence of new-onset cases. In addition, current evidence does not indicate a COVID-19 induced increase in the incidence of type 1 diabetes. A survey of 216 pediatric diabetes centers in Germany found no increase in incidence of type 1 diabetes during the COVID-19 pandemic compared to the previous nine years (42, 43). Although the same group did report an increase in incidence of diabetic ketoacidosis in new-onset cases and suggested that this was due to delayed admission to the health care system (44). Other studies have reported that the presentation of new-onset childhood diabetes was more severe during the pandemic with a large increase in those presenting with diabetic ketoacidosis and suggested that this was related to changes in parental behavior and access to healthcare (45–48). In general, there are still many uncertainties; including the extent that COVID-19 can induce or promote type 1 diabetes, type 2 diabetes or indeed an atypical form of diabetes. To address the many unknowns, an international group of leading diabetes researchers established the CoviDIAB Project with a global registry of patients with Covid-19–related diabetes (covidiab.e-dendrite.com) (49).

## Mucormycosis

The other COVID-associated pandemic is mucormycosis, a normally rare infection caused by a fairly ubiquitous filamentous mold fungus (50). However, large numbers of cases have been reported in patients with COVID-19 (51): as with COVID-19, males appear to be at greater risk (52). Predominantly the cases have been within India, which reported over 45,000 cases and 4,300 deaths between April and July of 2021 (51); but cases have also been reported globally (52). The infections have mostly been rhino-orbital mucormycosis which has a mortality rate as high as 30-50% with limited treatment options frequently necessitating the removal of the eyes (52, 53). Prior to COVID-19 it had been established that infection with mucormycosis was facilitated by a combination of conditions including being immunocompromised, hyperglycemia and acidosis, particularly ketoacidosis (54). The report from Logette E et al. (5) reveals at the atomistic level that the hyperglycemia associated with COVID-19 creates these very same conditions in the upper airway that are ideal for mucormycetes to invade.

Mucormycosis infection proceeds *via* an interaction between a fungal spore coat protein and cell surface receptors in the nasal passage that have been identified as glucose-regulated protein 78 (GRP78) (52, 54). GRP78 is as a protein chaperone that facilitates proteins entering the cell *via* endocytosis (55, 56); its abundance is induced by glycemic stress (57, 58) and inflammatory cytokines stimulate its translocation to the cell surface (59). A role for GRP78 in COVID-19 had already been implicated with evidence that the SARS-CoV-2 Spike-protein binds to GRP78 and that viral entry and replication within host cells could be suppressed by inhibition (60) or depletion of GRP78 (61). Indeed, many viruses appear to hijack GRP78 to infect human cells (62). This common mechanism for cell invasion provides a link explaining the importance of hyperglycemia in both the COVID-19 and mucormycosis pandemics (51–53) and demonstrates the broader implications of the AI generated insights into how hyperglycemia affects the internal milieu (5).

## Summary

The intense research activity surrounding COVID-19 has also accelerated our understanding of how metabolism affects infectious diseases; the extensive review by the work of Logette and colleagues (5) nicely brings this together We are now more aware of how hyperglycemia impacts a multitude of mechanisms that are involved in the body’s response to infections and the wider implications that open up new avenues of research. Undoubtedly COVID-19 will not be the last pandemic and this new information may prove invaluable for how these may be managed clinically in the future.

## Author Contributions

The author confirms being the sole contributor of this work and has approved it for publication.

## Conflict of Interest

The author declares that the research was conducted in the absence of any commercial or financial relationships that could be construed as a potential conflict of interest.

## Publisher’s Note

All claims expressed in this article are solely those of the authors and do not necessarily represent those of their affiliated organizations, or those of the publisher, the editors and the reviewers. Any product that may be evaluated in this article, or claim that may be made by its manufacturer, is not guaranteed or endorsed by the publisher.

## References

[1] van Crevel R van de Vijver S Moore DAJ. The Global Diabetes Epidemic: What Does it Mean for Infectious Diseases in Tropical Countries? Lancet Diabetes Endocrinol (2017) 5(6):457–68. doi: 10.1016/S2213-8587(16)30081-X PMC7104099 27499355

[2] Kickbusch I Leung GM Bhutta ZA Matsoso MP Ihekweazu C Abbasi K. Covid-19: How a Virus is Turning the World Upside Down. BMJ (2020) 369:m1336. doi: 10.1136/bmj.m1336 32245802

[3] Holly JMP Biernacka K Maskell N Perks CM. Obesity, Diabetes and COVID-19: An Infectious Disease Spreading From the East Collides With the Consequences of an Unhealthy Western Lifestyle. Front Endocrinol (Lausanne) (2020) 11:582870. doi: 10.3389/fendo.2020.582870 33042029 PMC7527410

[4] Muniangi-Muhitu H Akalestou E Salem V Misra S Oliver NS Rutter GA. Covid-19 and Diabetes: A Complex Bidirectional Relationship. Front Endocrinol (Lausanne) (2020) 11:582936. doi: 10.3389/fendo.2020.582936 33133024 PMC7578412

[5] Logette E Lorin C Favreau CPH Oshurko E Coggan JS Casalegno F. Elevated Blood Glucose Levels as a Primary Risk Factor for the Severity of COVID-19. Front Public Health (2021) 9:5. doi: 10.3389/fpubh.2021.695139 PMC8356061 34395368

[6] Drucker DJ. Diabetes, Obesity, Metabolism, and SARS-CoV-2 Infection: The End of the Beginning. Cell Metab (2021) 33(3):479–98. doi: 10.1016/j.cmet.2021.01.016 PMC7825982 33529600

[7] Nakhleh A Shehadeh N. Glycemic Control of Type 2 Diabetic Patients With Coronavirus Disease During Hospitalization: A Proposal for Early Insulin Therapy. Am J Physiol Endocrinol Metab (2020) 318:E835–7. doi: 10.1152/ajpendo.00163.2020 PMC7237499 32401039

[8] Valk T McMorrow C. Managing Hyperglycemia During the COVID-19 Pandemic: Improving Outcomes Using New Technologies in Intensive Care. SAGE Open Med (2020) 8:2050312120974174. doi: 10.1177/2050312120974174 33282306 PMC7686601

[9] Yu B Li C Sun Y Wang DW. Insulin Treatment is Associated With Increased Mortality in Patients With COVID-19. Cell Metab (2020) 33(1):65–77.e2. doi: 10.1016/j.cmet.2020.11.014 33248471 PMC7682421

[10] Donath MY. Glucose or Insulin, Which Is the Culprit in Patients With COVID-19 and Diabetes? Cell Metab (2021) 33(1):2–4. doi: 10.1016/j.cmet.2020.11.015 33248018 PMC7685630

[11] Gunst J De Bruyn A Van den Berghe G. Glucose Control in the ICU. Curr Opin Anaesthesiol (2019) 32:156–62. doi: 10.1097/ACO.0000000000000706 PMC6774765 30817388

[12] Carr MJ Wright AK Leelarathna L Thabit H Milne N Kanumilli N. Impact of COVID-19 on Diagnoses, Monitoring, and Mortality in People With Type 2 Diabetes in the UK. Lancet Diabetes Endocrinol (2021) 9(7):413–5. doi: 10.1016/S2213-8587(21)00116-9 PMC8112824 33989537

[13] Holman N Knighton P Kar P O'Keefe J Curley M Weaver A. Risk Factors for COVID-19-Related Mortality in People With Type 1 and Type 2 Diabetes in England: A Population-Based Cohort Study. Lancet Diabetes Endocrinol (2020) 8(10):823–33. doi: 10.1016/S2213-8587(20)30271-0 PMC7426091 32798471

[14] Al Mahmeed W Al-Rasadi K Banerjee Y Ceriello A Cosentino F Galia M. Promoting a Syndemic Approach for Cardiometabolic Disease Management During COVID-19: The CAPISCO International Expert Panel. Front Cardiovasc Med (2021) 8:787761. doi: 10.3389/fcvm.2021.787761 34977193 PMC8715947

[15] NICE. Overview COVID-19 Rapid Guideline: Managing the Long-Term Effects of COVID-19 Guidance, 2020. Available at: https://www.nice.org.uk/guidance/ng188 (Accessed 05 May 2021).

[16] WHO. A Clinical Case Definition of Post COVID-19 Condition by a Delphi Consensus. Oct 6, 2021. Geneva: World Health Organization (2021).

[17] Briggs A Vassall A. Count the Cost of Disability Caused by COVID-19. Nature (2021) 593(7860):502–5. doi: 10.1038/d41586-021-01392-2 34040204

[18] PHOSP-COVID Collaborative Group. Physical, Cognitive and Mental Health Impacts of COVID-19 Following Hospitalisation – A Multi-Centre Prospective Cohort Study. medRxiv (2021). doi: 10.1101/2021.03.22.21254057

[19] Xie Y Bowe B Al-Aly Z. Burdens of Post-Acute Sequelae of COVID-19 by Severity of Acute Infection, Demographics and Health Status. Nat Commun (2021) 12(1):6571. doi: 10.1038/s41467-021-26513-3 34772922 PMC8589966

[20] Prescott HC Girard TD. Recovery From Severe COVID-19: Leveraging the Lessons of Survival From Sepsis. JAMA (2020) 324(8):739–40. doi: 10.1001/jama.2020.14103 32777028

[21] Gritte RB Souza-Siqueira T Curi R Machado MCC Soriano FG. Why Septic Patients Remain Sick After Hospital Discharge? Front Immunol (2021) 11:605666. doi: 10.3389/fimmu.2020.605666 33658992 PMC7917203

[22] Nalbandian A Sehgal K Gupta A Madhavan MV McGroder C Stevens JS. Post-Acute COVID-19 Syndrome. Nat Med (2021) 27:601–15. doi: 10.1038/s41591-021-01283-z PMC8893149 33753937

[23] Donath MY Meier DT Böni-Schnetzler M. Inflammation in the Pathophysiology and Therapy of Cardiometabolic Disease. Endocr Rev (2019) 40:1080–91. doi: 10.1210/er.2019-00002 PMC6624792 31127805

[24] Ayoubkhani D Khunti K Nafilyan V Maddox T Humberstone B Diamond I. Post-Covid Syndrome in Individuals Admitted to Hospital With Covid-19: Retrospective Cohort Study. BMJ (2021) 372:n693. doi: 10.1136/bmj.n693 33789877 PMC8010267

[25] Daugherty SE Guo Y Heath K Dasmariñas MC Jubilo KG Samranvedhya J. SARS-CoV-2 Infection and Risk of Clinical Sequelae During the Post-Acute Phase: A Retrospective Cohort Study. medRxiv (2021). doi: 10.1101/2021.03.12.21253448 PMC8132065 34011492

[26] Estiri H Strasser ZH Brat GA Semenov YR Consortium for Characterization of COVID-19 by EHR (4CE) Patel CJ. Evolving Phenotypes of non-Hospitalized Patients That Indicate Long COVID. BMC Med (2021) 19(1):249. doi: 10.1186/s12916-021-02115-0 34565368 PMC8474909

[27] Maestre-Muñiz MM Arias Á Mata-Vázquez E Martín-Toledano M López-Larramona G Ruiz-Chicote AM. Long-Term Outcomes of Patients With Coronavirus Disease 2019 at One Year After Hospital Discharge. J Clin Med (2021) 10:2945. doi: 10.3390/jcm10132945 34209085 PMC8269002

[28] Akter F Mannan A Mehedi HMH Rob MA Ahmed S Salauddin A. Clinical Characteristics and Short Term Outcomes After Recovery From COVID-19 in Patients With and Without Diabetes in Bangladesh. Diabetes Metab Syndr (2020) 14:2031–8. doi: 10.1016/j.dsx.2020.10.016 PMC7575434 33113469

[29] Kamal M Omirah MA Hussein A Saeed H. Assessment and Characterisation of Post-COVID-19 Manifestations. Int J Clin Pract (2021) 75(3):e13746. doi: 10.1111/ijcp.13746 32991035 PMC7536922

[30] Montefusco L Ben Nasr M D’Addio F Loretelli C Rossi A Pastore I. Acute and Long-Term Disruption of Glycometabolic Control After SARS-CoV-2 Infection. Nat Metab (2021) 3:774–85. doi: 10.1038/s42255-021-00407-6 PMC9931026 34035524

[31] Xiang Y Chau CK-L Qiu J Rao S So H-C. Exploring Causal Relationships Between COVID-19 and Cardiometabolic Disorders: A Bi-Directional Mendelian Randomization Study. medRxiv (2021). doi: 10.1101/2021.03.20.21254008

[32] Unsworth R Wallace S Oliver NS Yeung S Kshirsagar A Naidu H. New-Onset Type 1 Diabetes in Children During COVID-19: Multicenter Regional Findings in the U.K. Diabetes Care (2020) 43(11):e170–1. doi: 10.2337/dc20-1551 32816997

[33] Steenblock C Richter S Berger I Barovic M Schmid J Schubert U. Viral Infiltration of Pancreatic Islets in Patients With COVID-19. Nat Commun (2021) 12(1):3534. doi: 10.1038/s41467-021-23886-3 34112801 PMC8192507

[34] Qadir MMF Bhondeley M Beatty W Gaupp DD Doyle-Meyers LA Fischer T. SARS-CoV-2 Infection of the Pancreas Promotes Thrombofibrosis and is Associated With New-Onset Diabetes. JCI Insight (2021) 6(16):e151551. doi: 10.1172/jci.insight.151551 PMC8410013 34241597

[35] Wu CT Lidsky PV Xiao Y Lee IT Cheng R Nakayama T. SARS-CoV-2 Infects Human Pancreatic β Cells and Elicits β Cell Impairment. Cell Metab (2021) 33(8):1565–1576.e5. doi: 10.1016/j.cmet.2021.05.013 34081912 PMC8130512

[36] Bhavya Pathak E Mishra R. Deciphering the Link Between Diabetes Mellitus and SARS-CoV-2 Infection Through Differential Targeting of microRNAs in the Human Pancreas. J Endocrinol Invest (2021) 20:1–14. doi: 10.1007/s40618-021-01693-3 PMC8527307 34669152

[37] Rodriguez-Calvo T Sabouri S Anquetil F Von Herrath MG. The Viral Paradigm in Type 1 Diabetes: Who are the Main Suspects? Autoimmun Rev (2016) 15(10):964–9. doi: 10.1016/j.autrev.2016.07.019 27491567

[38] Accili D. Can COVID-19 Cause Diabetes? Nat Metab (2021) 3(2):123–5. doi: 10.1038/s42255-020-00339-7 PMC8892570 33432203

[39] Ward H Flower B Garcia PJ Ong SWX Altmann DM Delaney B. Global Surveillance, Research, and Collaboration Needed to Improve Understanding and Management of Long COVID. Lancet (2021) 398(10316):2057–9. doi: 10.1016/S0140-6736(21)02444-2 PMC8580495 34774190

[40] Fernández-de-Las-Peñas C Torres-Macho J Elvira-Martínez CM Molina-Trigueros LJ Sebastián-Viana T Hernández-Barrera V. Obesity is Associated With a Greater Number of Long-Term Post-COVID Symptoms and Poor Sleep Quality: A Multicentre Case-Control Study. Int J Clin Pract (2021) 75(12):e14917. doi: 10.1111/ijcp.14917 34569684 PMC8646300

[41] Saeedi P Petersohn I Salpea P Malanda B Karuranga S Unwin N. Global and Regional Diabetes Prevalence Estimates for 2019 and Projections for 2030 and 2045: Results From the International Diabetes Federation Diabetes Atlas, 9^th^ Edition. Diabetes Res Clin Pract (2019) 157:107843. doi: 10.1016/j.diabres.2019.107843 31518657

[42] Tittel SR Rosenbauer J Kamrath C Ziegler J Reschke F Hammersen J. Did the COVID-19 Lockdown Affect the Incidence of Pediatric Type 1 Diabetes in Germany? Diabetes Care (2020) 43(11):e172–3. doi: 10.2337/dc20-1633 PMC7576433 32826282

[43] Kamrath C Rosenbauer J Tittel SR Warncke K Hirtz R Denzer C. Frequency of Autoantibody-Negative Type 1 Diabetes in Children, Adolescents, and Young Adults During the First Wave of the COVID-19 Pandemic in Germany. Diabetes Care (2021) 44(7):1540–6. doi: 10.2337/dc20-2791 33990377

[44] Kamrath C Rosenbauer J Eckert AJ Pappa A Reschke F Rohrer TR. Incidence of COVID-19 and Risk of Diabetic Ketoacidosis in New-Onset Type 1 Diabetes. Pediatrics (2021) 107:180–5. doi: 10.1542/peds.2021-050856 34011636

[45] Salmi H Heinonen S Hästbacka J Lääperi M Rautiainen P Miettinen PJ. New-Onset Type 1 Diabetes in Finnish Children During the COVID-19 Pandemic. Arch Dis Child (2021) 107:180–85. doi: 10.1136/archdischild-2020-321220 34045208

[46] McGlacken-Byrne SM Drew SEV Turner K Peters C Amin R. The SARS-CoV-2 Pandemic is Associated With Increased Severity of Presentation of Childhood Onset Type 1 Diabetes Mellitus: A Multi-Centre Study of the First COVID-19 Wave. Diabetes Med (2021) 38(9):e14640. doi: 10.1111/dme.14640 PMC8420510 34245598

[47] DiMeglio LA Albanese-O'Neill A Muñoz CE Maahs DM. COVID-19 and Children With Diabetes-Updates, Unknowns, and Next Steps: First, Do No Extrapolation. Diabetes Care (2020) 43(11):2631–4. doi: 10.2337/dci20-0044 32887703

[48] Elbarbary NS Dos Santos TJ de Beaufort C Agwu JC Calliari LE Scaramuzza AE. Pediatr COVID-19 Outbreak and Pediatric Diabetes: Perceptions of Health Care Professionals Worldwide. Diabetes (2020) 21(7):1083–92. doi: 10.1111/pedi.13084 PMC7404589 32686287

[49] Rubino F Amiel SA Zimmet P Alberti G Bornstein S Eckel RH. New-Onset Diabetes in Covid-19. N Engl J Med (2020) 383(8):789–90. doi: 10.1056/NEJMc2018688 PMC7304415 32530585

[50] Spellberg B Edwards J Jr Ibrahim A. Novel Perspectives on Mucormycosis: Pathophysiology, Presentation, and Management. Clin Microbiol Rev (2005) 18:556–69. doi: 10.1128/CMR.18.3.556-569.2005 PMC1195964 16020690

[51] Divakar PK. Fungal Taxa Responsible for Mucormycosis/"Black Fungus" Among COVID-19 Patients in India. J Fungi (Basel) (2021) 7:641. doi: 10.3390/jof7080641 34436180 PMC8402169

[52] Pasrija R Naime M. Resolving the Equation Between Mucormycosis and COVID-19 Disease. Mol Biol Rep (2022) 22:1–8. doi: 10.1007/s11033-021-07085-3 PMC8782700 35064406

[53] Pal R Singh B Bhadada SK Banerjee M Bhogal RS Hage N. COVID-19-Associated Mucormycosis: An Updated Systematic Review of Literature. Mycoses (2021) 64:1452–9. doi: 10.1111/myc.13338 PMC8447126 34133798

[54] Baldin C Ibrahim AS. Molecular Mechanisms of Mucormycosis-The Bitter and the Sweet. PloS Pathog (2017) 13(8):e1006408. doi: 10.1371/journal.ppat.1006408 28771587 PMC5542377

[55] Lee AS. The ER Chaperone and Signaling Regulator GRP78/BiP as a Monitor of Endoplasmic Reticulum Stress. Methods (2005) 35:373–81. doi: 10.1016/j.ymeth.2004.10.010 15804610

[56] Merkel A Chen Y George A. Endocytic Trafficking of DMP1 and GRP78 Complex Facilitates Osteogenic Differentiation of Human Periodontal Ligament Stem Cells. Front Physiol (2019) 10:1175. doi: 10.3389/fphys.2019.01175 31572220 PMC6751249

[57] Li J Ni M Lee B Barron E Hinton DR Lee AS. The Unfolded Protein Response Regulator GRP78/BiP is Required for Endoplasmic Reticulum Integrity and Stress-Induced Autophagy in Mammalian Cells. Cell Death Differ (2008) 15:1460–71. doi: 10.1038/cdd.2008.81 PMC2758056 18551133

[58] Ibrahim IM Abdelmalek DH Elfiky AA. GRP78: A Cell’s Response to Stress. Life Sci (2019) 226:156–63. doi: 10.1016/j.lfs.2019.04.022 PMC7094232 30978349

[59] Vig S Buitinga M Rondas D Crevecoeur I van Zandvoort M Waelkens E. Cytokine-Induced Translocation of GRP78 to the Plasma Membrane Triggers a Pro-Apoptotic Feedback Loop in Pancreatic Beta Cells. Cell Death Dis (2019) 10:309. doi: 10.1038/s41419-019-1518-0 30952835 PMC6450900

[60] Rayner JO Roberts RA Kim J Poklepovic A Roberts JL Booth L. AR12 (OSU-03012) Suppresses GRP78 Expression and Inhibits SARS-CoV-2 Replication. Biochem Pharmacol (2020) 182:114227. doi: 10.1016/j.bcp.2020.114227 32966814 PMC7502229

[61] Carlos AJ Ha DP Yeh DW Van Krieken R Tseng CC Zhang P. The Chaperone GRP78 is a Host Auxiliary Factor for SARS-CoV-2 and GRP78 Depleting Antibody Blocks Viral Entry and Infection. J Biol Chem (2021) 6:100759. doi: 10.1016/j.jbc.2021.100759 PMC8102082 33965375

[62] Gonzalez-Gronow M Gopal U Austin RC Pizzo SV. Glucose -Regulated Protein (GRP78) is an Important Cell Surface Receptor for Viral Invasion, Cancers and Neurological Disorders. IUBMB Life (2021) 73:843–54. doi: 10.1002/iub.2502 33960608

